# Clinical Significance of Psychiatric Comorbidities Among Outpatients With Gambling Disorder in Japan: A 12‐Month Follow‐Up Study

**DOI:** 10.1002/npr2.70016

**Published:** 2025-05-01

**Authors:** Risa Yamada, Andrew Stickley, Masahiro Shigeta, Hisatsugu Miyata

**Affiliations:** ^1^ Department of Preventive Intervention for Psychiatric Disorders National Institute of Mental Health, National Center of Neurology and Psychiatry Tokyo Japan; ^2^ Department of Psychiatry The Jikei University School of Medicine Tokyo Japan; ^3^ Hirakawa Hospital Tokyo Japan

**Keywords:** abstinence, behavioral addictions, comorbidity, gambling disorder, prospective study

## Abstract

**Background:**

Gambling disorder (GD) is often comorbid with other psychiatric disorders. Previous studies have reported that psychiatric comorbidity increases both treatment dropout and relapse among patients with GD. However, little is known about the effects of comorbidity among outpatients with GD either during or after treatment. This study examined this issue in a clinical setting.

**Method:**

60 outpatients with GD (men/women, 58/2; average age, 37.9 years) participated in assessments of the course of multidimensional treatment outcomes (i.e., gambling‐related variables, social impairment, and depressive symptoms) at three time points (baseline, 3 months, and 12 months). We examined treatment outcomes in GD patients with and without comorbidity.

**Results:**

A total of 58.3% of the patients had comorbidities (major depressive disorder, 26.7%; behavioral addiction, 21.7%; anxiety disorder, 15.0%, etc.), while the dropout rates across the study period were 13.3% at 3 months and 35.0% at 12 months. Statistical analyses indicated that the South Oaks Gambling Screen score and the Sheehan Disability Scale score were significantly reduced at follow‐up compared to baseline in both comorbid and non‐comorbid outpatients. There was a significant difference for being still in treatment, where the proportion of individuals with comorbid disorders was significantly higher than those without comorbidities at the 12‐month compared to the 3‐month follow‐up. The proportion of all outpatients who remained abstinent from gambling at 12 months was significantly lower compared to baseline and at 3 months.

**Conclusions:**

Being still in treatment for comorbid psychiatric problems may affect the course and outcome of GD treatment.

## Abstract 2

This study examined the prognosis of GD in outpatients based on comorbidity status in Japan. Surprisingly, psychiatric comorbidity was not linked to worse GD outcomes. Continued treatment for comorbidities may impact GD treatment outcomes and help prevent adverse effects.

## Graphical abstract

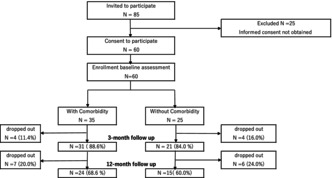

## 1 Introduction

Gambling disorder (GD) is characterized by maladaptive patterns of gambling that persist despite the fact that they can lead to serious health consequences, and financial and legal problems [1]. GD is a global public health concern, with an earlier systematic review showing that the past 12‐month rates of problematic gambling ranged between 0.12% and 5.8% worldwide [5]. In Japan, the lifetime prevalence of problematic gambling has been estimated as being 3.8%, while for the past 12 months it is 0.8%, suggesting that gambling is also a significant concern in this setting [15].

GD patients also often have psychiatric comorbidities. In particular, results from a meta‐analysis showed that 74.8% of patients seeking treatment for GD had other psychiatric disorders [7]. This finding was echoed in a Japanese study where 58.3% of GD patients had one or more comorbid psychiatric disorders (tobacco use disorder, 20.9%; alcohol use disorder, 13.9%; major depressive disorder, 13.1%; behavioral addictions, 13.1%; attention‐deficit hyperactivity disorder [ADHD] 6.1%) [33]. Having a comorbid psychiatric disorder tends to worsen the outcomes associated with GD when compared to non‐comorbid cases. For example, the results from a large mixed sample of pathological gamblers (recruited via the general population, at gambling locations, through a project telephone hotline, and among in‐patients being treated for pathological gambling) showed that mood disorders (relative risk ratio, RRR = 11.92) and Cluster B personality disorders (RRR = 2.40) were associated with suicidal outcomes [2]. Furthermore, other research has also indicated that the presence of comorbid conditions such as substance use disorders, depression, anxiety, and ADHD results in a greater gambling severity than among participants with non‐comorbid gambling problems [10]. In addition, a recent study also indicated that individuals with GD that was comorbid with behavioral addictions including kleptomania, excessive buying, and excessive sex‐related behavior may have more severe gambling problems than those without such behavioral addictions [33].

Psychopathological comorbidity in individuals with GD is thought to affect access and adherence to treatment and may therefore negate the effectiveness of psychological interventions [31]. Several studies have explored the association between treatment course outcomes and psychiatric comorbidity among patients with GD. In a recent study from Germany that focused on outpatient gambling care, although all of the patients benefited from care, the improvement in GD severity was poorer in patients with anxiety disorders compared to those without this comorbidity [32]. In a prospective study that used a naturalistic sample of individuals with GD who had recently stopped gambling, those with no history of drug abuse or dependence were over 2.5 times more likely to have achieved at least 3 months of abstinence from gambling compared to those with a lifetime drug disorder diagnosis, while individuals who had experienced a mood disorder during their lives were 46% less likely to experience 3‐month abstinence compared to individuals with no history of mood disorder [16]. A prior study that examined the predictors of early GD treatment dropout highlighted those specific forms of psychiatric comorbidity (e.g., having an antisocial personality disorder, drug dependence, and posttraumatic stress disorder) that were strongly associated with treatment failure in a large sample of treatment‐seeking patients with GD [24]. Thus, the presence of psychiatric comorbidity may have a negative impact on treatment outcomes (e.g., abstinence, treatment dropout and increased GD severity).

Until now, most previous prospective studies on treatment outcomes in GD have only measured gambling problems (e.g., abstinence, the severity of GD) despite the high prevalence of psychiatric comorbidity in individuals with GD and its demonstrated detrimental association with GD [16, 24, 32]. Moreover, some reviews of treatment outcomes have highlighted that to gain a better understanding of treatment progress in individuals with GD, it is necessary to evaluate a wide range of outcome domains such as gambling behavior (e.g., the frequency of gambling in days per month, the severity of GD) and the problems caused by gambling (e.g., impaired social functioning, and mental health problems) [4, 28, 30]. The incorporation of broader outcome domains that extend beyond disorder‐specific symptoms and behaviors would allow the efficacy of an intervention to be assessed in a variety of areas of functioning (e.g., work, family, and social functioning) in GD patients with psychiatric comorbidities. Additionally, to our knowledge, as yet, there has been no naturalistic prospective research on GD patients with psychiatric comorbidities in clinical settings in Japan.

Thus, the objective of this study is to investigate whether multifaceted treatment outcomes (i.e., the severity of GD, abstinence of gambling, treatment participation, social functioning, and the severity of depression) differ between GD patients with and without comorbid psychiatric conditions in a clinical setting.

## 2 Methods

### 2.1 Participants and Procedure

**FIGURE 1:**
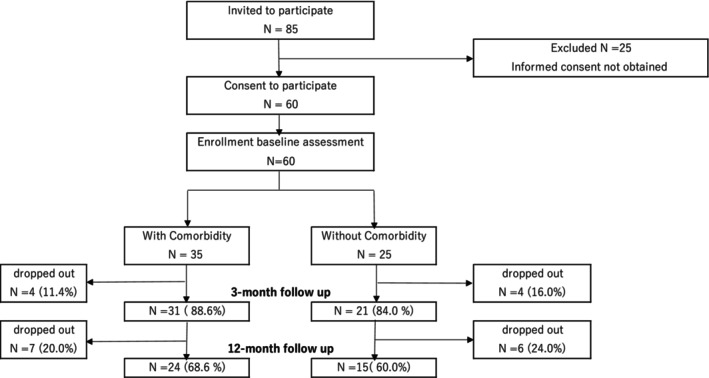
Flowchart of this study.

Participants comprised 60 GD outpatients who received their first medical examinations during the period from April 2020 through March 2022 at one of 10 institutions for treating addictions, including nine clinics and one hospital (located in Aichi, Fukuoka, Kanagawa, Osaka, Tokyo). Recruitment was initiated by research staff contacting these medical institutions with information about the study. Individuals were excluded from participating if: (1) they were 19 years old or younger, (2) there had not been a GD diagnosis made by the doctor in charge using DSM‐5 criteria, and (3) the patient was hospitalized (including being scheduled to go into hospital). Participants were given an information sheet about the study and asked to provide written informed consent before the data collection began (agreement rate: 70.6%) (see Figure 1).

A prospective cohort study was undertaken where participants were assessed at three time points (baseline, at 3 months, and at 12 months from baseline). The evaluation at baseline occurred within a week of the first medical examination.

The recruitment period ran from April 2020 through March 2022. The final follow‐up was completed in March 2023. To improve response rates [9], self‐report questionnaires were administered by mail, and mail‐outs included a gift voucher of 5000 yen on return of the baseline, 3‐month, and 12‐month questionnaires. The research staff also followed up on these mail‐outs by contacting those who had not returned the questionnaires. This study was approved by the Ethics Committee at the Jikei University School of Medicine (31‐443 (10025)).

### 2.2 Assessment of Background and Clinical Information

This study examined patients' sociodemographic and clinical characteristics and gambling problems through the use of self‐reports. Sociodemographic characteristics included sex, age, education (completed junior high school, high school or technical school, college), marital status (married, divorced, never married), employment status (salaried job, daily wage, student, unemployed), and adverse childhood experiences (e.g., economic poverty, bullying experiences). Examples of gambling problems included information on the type of gambling activity (e.g., pachinko: Japanese pinball, pachislo: Japanese slot machines, horse racing, or lottery), age of gambling onset, the frequency of gambling (days of gambling per month), and the reason for seeking treatment. In addition, information was also obtained on current and past medical/psychiatric history, history of social support (participation in gambling self‐help groups), and history of psychiatric treatment for GD such as using medication, attending group meetings, counseling, and undergoing cognitive behavioral therapy. In this study, group meetings and counseling were defined as not including an element of cognitive behavioral therapy.

### 2.3 Clinical Assessments via a Self‐Report Questionnaire

The Sheehan Disability Scale (SDS), a self‐report instrument, was used to assess social disability. Its three items are grouped into three categories: functioning in relation to (i) work, (ii) social or leisure activities, and (iii) home and family life. Items are rated using a 10‐point Likert scale ranging from 0 (unimpaired) to 10 (highly impaired) over the last 2 weeks. Total scores range from 0 to 30, with higher scores indicating increased impairment [22]. This study used the Japanese version of the SDS [34].

The Patient Health Questionnaire‐9 (PHQ‐9) is a 9‐item instrument given to patients in primary care settings to screen for the presence and severity of depression according to DSM‐criteria over the last 2 weeks. Response options relating to the frequency of symptoms range from “not at all” (scored 0) to “nearly every day” (3). Eight items assess depressive symptoms while the final item is focused on suicidal ideation. Total scores range from 0 to 27 with higher scores indicating more depression symptoms. A threshold score of 10 or higher is considered to indicate “moderate” depression, 15 or higher indicates “moderately severe” depression, and 20 or higher “severe” depression [20]. This study used the Japanese version of the PHQ‐9 [26] with a cut‐off score of 10 being used to categorize cases.

The South Oaks Gambling Screen (SOGS) is a 20‐item questionnaire that measures gambling behavior with questions that focus on the participant's history of gambling, the frequency at which the person engages in these behaviors, and obstacles that gambling may have posed in the participant's life assessed with questions with yes/no answer options. The score on the SOGS ranges from 0 to 20; a score of 5 and above indicates probable GD [23]. This study used the Japanese version of the SOGS [18].

### 2.4 Assessments of Psychiatric Disorders

Psychiatric disorders were assessed by the patients' doctors using DSM‐5 criteria. Additionally, our research team under the supervision of the senior researcher (H.M.), reassessed the psychiatric disorders based on the information from the self‐reports and patients' doctors using DSM‐5 criteria.

Furthermore, in this study, kleptomania, excessive buying behavior, excessive sex‐related behavior, and internet gaming disorder were categorized as behavioral addictions. Although DSM‐5 classifies GD as an addictive disorder, as yet, the other types of behavior mentioned above have not been classified as addictive disorders because sufficient evidence has not yet been accumulated to do this. Nonetheless, some studies [6, 27] have categorized kleptomania [12], excessive buying behavior [25], excessive sex‐related behaviors [11], and internet gaming disorder [1] as behavioral addictions based on their symptomatologic similarities. Therefore, these four types of behavior were considered to constitute behavioral addictions in this study. In relation to this, Griffiths' behavioral addiction model was used in the current study to make a diagnosis of excessive buying and excessive sex‐related behaviors because DSM‐5 has no diagnostic criteria for these two disorders. The structure of Griffiths' behavioral addiction model is similar to that of substance dependence and GD in DSM‐5 in that it includes craving, loss of control, tolerance, withdrawal, and social disturbance as criteria [14]. As with the other psychiatric disorders mentioned above, our research team also made a reassessment of these behavioral addictions based on the information from the self‐reports and patients' doctors under the supervision of the senior researcher (H.M.).

### 2.5 Outcome Measures

Outcome status was represented by current sociodemographic and clinical characteristics (e.g., marital status, employment), treatment status (e.g., still in treatment or not), social support (participation in self‐help groups or not), gambling problems (continue to engage in gambling activities or not, category of gambling activities, and the number of days of participating in gambling activities over a month), as well as several self‐administered questionnaires (the PHQ‐9, SDS, SOGS). As with the original versions, the Japanese versions of the PHQ‐9 and SDS evaluated outcomes over the last 2 weeks. Meanwhile, the SOGS asked about gambling problems from the previous evaluated point until now.

### 2.6 Statistical Analysis

For the sample characteristics, the means and SDs of the continuous variables were calculated, as were the frequencies and proportions of the categorical variables. Univariable analyses (using Fisher's exact test or Pearson's chi‐squared test for categorical variables and *t* tests for continuous variables) were conducted to compare psychiatric diagnoses grouped according to comorbidity status (i.e., by the non‐comorbidity and the comorbidity groups). Given the longitudinal, repeated measures study design, generalized estimating equations (GEE) were used for categorical variables, and generalized linear mixed models (GLMM) for continuous variables to detect significant changes over time.

All *p* values were two‐sided; *p* < 0.05 was considered statistically significant. All statistical analyses were conducted using IBM SPSS Statistics software (version 29.0; SPSS Japan Inc.).

## 3 Results

### 3.1 Sociodemographic and Clinical Characteristics of Patients With GD

Of the 60 patients included at baseline, 86.7% (*n* = 52) participated at the 3‐month follow‐up, and 65.0% (*n* = 39) at the 12‐month follow‐up. Drop‐out rates across the study period were 13.3% at the 3‐month point (11.4% among patients with psychiatric comorbidities and 16.0% among patients without comorbidities), and 35.0% at 12 months (31.4% with comorbidities and 40.0% without comorbidities).

The sociodemographic and clinical characteristics of the patients with GD stratified by psychiatric comorbidity status at baseline are presented in Table 1. The average age of the patients with GD was 37.9 years In terms of education, most patients had completed high school (41.7%), while one‐third had a college education. Almost half of the patients (48.3%) were married, whereas 38.3% had never been married. The majority of patients had a salaried job or received a daily wage (70.0%) although one‐quarter were unemployed. In terms of childhood adversities, just under one‐third of the patients (30.0%) had experienced economic poverty in childhood, while over 20% had been bullied. Over one‐quarter (28.3%) of the patients had a history of participation in self‐help groups. Regarding psychiatric treatment for GD, over one‐third of the patients had participated in group meetings (33.3%), 28.3% had undergone counseling, while almost one in 10 had received cognitive behavior therapy (8.3%). The sociodemographic and clinical characteristics of the participants in the “With comorbidity” and “Without comorbidity” groups are also shown in Table 1. There were no significant between‐group differences.

**TABLE 1:** Sociodemographic and clinical characteristics of participants with or without psychiatric comorbidity at baseline.

	Psychiatric comorbidity						
	All patients (*n* = 60)		With comorbidity (*n* = 35)		Without comorbidity (*n* = 25)		*p*
Male (*n*, %)	58	96.7%	33	94.3%	25	100.0%	0.506
Age (average, SD)	37.9	9.35	38.7	9.43	36.7	9.30	0.430
Education (%)							
≥ Completed college	21	35.0%	9	25.7%	12	48.0%	0.198
Completed technical school	11	18.3%	7	20.0%	4	16.0%	
Completed high school	25	41.7%	16	45.7%	9	36.0%	
Completed junior high school	3	5.0%	3	8.6%	0	0.0%	
Martial state (*n*, %)							
Married	29	48.3%	17	48.6%	12	48.0%	0.391
Divorced	8	13.3%	3	8.6%	5	20.0%	
Never married	23	38.3%	15	42.9%	8	32.0%	
Employment status (*n*, %)							
Salaried job	36	60.0%	18	51.4%	18	72.0%	0.445
Daily wage	6	10.0%	4	11.4%	2	8.0%	
Student	3	5.0%	2	5.7%	1	4.0%	
Unemployed	15	25.0%	11	31.4%	4	16.0%	
Adverse childhood experiences							
Economic poverty	18	30.0%	13	37.1%	5	20.0%	0.253
Bullying	14	23.3%	11	31.4%	3	12.0%	0.122
Poor grades	8	13.3%	6	17.1%	2	8.0%	0.449
Strict upbringing	8	13.3%	5	14.3%	3	12.0%	1.000
Excessive expectations	8	13.3%	5	14.3%	3	12.0%	1.000
Comorbid psychiatric disorders							
Major depressive disorder	16	26.7%	16	47.1%			
Behavioral addictions (Kleptomania, shopping, sex‐related)	13	21.7%	13	38.2%			
Anxiety disorder	9	15.0%	9	26.5%			
Intellectual disabilities	3	5.0%	3	8.8%			
Alcohol use disorder	3	5.0%	3	8.8%			
Bipolar disorder	2	3.3%	2	5.9%			
Dissociative disorder	1	1.7%	1	2.9%			
History of medical treatment							
Psychiatric medication (current)	6	10.0%	5	14.3%	1	4.0%	0.386
Group meetings	20	33.3%	11	31.4%	9	36.0%	0.785
Counseling	17	28.3%	11	31.4%	6	24.0%	0.575
Cognitive behavior therapy	5	8.3%	4	11.4%	1	4.0%	0.390
History of participating in self‐help groups (*n*, %)	17	28.3%	10	28.6%	7	28.0%	1.000

*Note:* *p* value for *t*‐test (continuous variable). *p* value for Pearson's chi‐squared test or Fisher's exact test (categorical variables).

Among the 60 patients, 35 (58.3%) had some form of psychiatric comorbidity. As shown in Table 1, a number of psychiatric disorders were comorbid with GD. The percentage of each comorbid disorder ranged from 2.9% (dissociative disorder) up to 47.1% (major depressive disorder) among those with comorbidities. There was one patient in the “Without Comorbidity” group who had been diagnosed with only GD by the doctor in charge. The patient also self‐reported no comorbid psychiatric disorders, despite appearing to be taking psychiatric medication. To ensure accuracy, the senior researcher (H.M.) reassessed the patient's psychiatric status based on self‐reported information and communication with the doctor in charge, using DSM‐5 criteria. Based on this evaluation, the patient was ultimately classified as being “Without Comorbidity.”

Gambling‐related variables among individuals with and without psychiatric comorbidity at baseline are shown in Table 2. The average age of gambling activities onset was 19.5 years, while the average age when engaging in high‐frequency gambling activities was 30.3 years. The average number of gambling days in the past month was 22.5. The most popular form of gambling was pachinko (a Japanese pinball game; 80.0%), followed by pachislo (a Japanese slot machine; 66.7%), and betting on horse racing (55.0%). In terms of gambling‐related problems, suicidal ideation was prevalent (78.3%), while 11.7% of the patients had made a lifetime suicide attempt.

**TABLE 2:** Gambling‐related variables among outpatients with or without psychiatric comorbidity at baseline.

	Psychiatric comorbidity						
	Patient totals (*n* = 60)		With comorbidity (*n* = 35)		Without comorbidity (*n* = 25)		*p*
Age of gambling onset (average, SD)	19.5	3.93	20.2	4.64	18.6	2.42	0.106
Period of heavy gambling							
Age (average, SD)	30.3	9.10	30.6	8.39	29.8	10.2	0.762
Gambling days in the last 30 days (average, SD)	22.5	7.61	23.4	7.50	21.3	7.75	0.298
Gambling activity (multiple answers) (*n*, %)							
Pachinko	48	80.0%	28	80.0%	20	80.0%	1.000
Pachislo	40	66.7%	24	68.6%	16	64.0%	0.785
Horse racing	33	55.0%	17	48.6%	16	64.0%	0.297
Boat racing	21	35.0%	14	40.0%	7	28.0%	0.416
Amusement arcade	18	30.0%	12	34.3%	6	24.0%	0.569
Lottery	17	28.3%	13	37.1%	4	16.0%	0.089
Bicycle racing	16	26.7%	7	20.0%	9	36.0%	0.238
Gambling‐related problems (*n*, %)							
Suicidal ideation	47	78.3%	28	80.0%	19	76.0%	0.758
Disappearing because of gambling debts	12	20.0%	9	25.7%	3	12.0%	0.327
Suicide attempt	7	11.7%	6	17.1%	1	4.0%	0.222

*Note:* *p* value for *t*‐test (continuous variables). *p* value for Pearson's chi‐squared test or Fisher's exact test (categorical variables).

### 3.2 Outcome Measures

Tables 3 and 4 show the results of all the treatment outcomes for the GD patients at the three time points (baseline, 3 months, and 12 months from baseline).

**TABLE 3:** The course of gambling disorder (gambling problems, depressive symptoms, and social dysfunction) among outpatients at the three time points.

	With comorbidity (*n* = 35)		Without comorbidity (*n* = 25)		Time effect		Summary of significant differences	Time × group interaction	
		SD		SD	*F*	*p*		*F*	*p*
*(1) Gambling days last 30 days*									
a. Baseline	3.37	7.52	0.40	2.00	0.223	0.801	*a* = *b* = *c*	1.310	0.275
b. 3 months	1.74	5.02	1.00	4.36					
c. 12 months	3.42	6.12	0.33	1.05					
*(2) SOGS*									
a. Baseline	13.57	2.88	13.40	2.53	70.98	**< 0.001**	*a* > *b*, c**	1.69	0.190
b. 3 months	5.71	5.40	3.67	5.29					
c. 12 months	7.63	6.07	4.47	6.30					
*(3) PHQ‐9*									
a. Baseline	9.37	7.55	7.00	5.37	4.46	**0.014**	*a* > c*	0.27	0.763
b. 3 months	8.58	5.90	4.81	4.20					
c. 12 months	7.46	5.41	4.07	3.01					
*(4) SDS work*									
a. Baseline	3.86	3.57	2.40	2.65	8.66	**< 0.001**	*a* > *b*, c**	0.35	0.709
b. 3 months	2.27	2.63	0.90	1.48					
c. 12 months	2.04	2.54	1.20	1.66					
*Social or leisure activities*									
a. Baseline	3.09	3.33	2.68	2.77	5.29	**0.007**	*a* > *b*, c*	0.55	0.578
b. 3 months	2.57	2.40	1.38	1.86					
c. 12 months	2.17	2.24	1.07	1.58					
*Home and family life*									
a. Baseline	3.89	3.72	3.80	3.22	10.1	**< 0.001**	*a* > *b*, c**	0.94	0.394
b. 3 months	2.53	3.39	1.24	2.30					
c. 12 months	2.38	2.60	1.00	1.41					
*Total score*									
a. Baseline	10.83	8.72	8.88	7.48	11.81	**< 0.001**	*a* > b, c**	0.38	0.685
b. 3 months	7.37	7.03	3.52	4.30					
c. 12 months	6.58	6.34	3.27	4.42					

*Note:* *p* value for generalized linear mixed models (*p < 0.05, **p < 0.01). Significant *p* values are shown in bold font (*p* < 0.05).

Abbreviations: SD, standard deviation; SDS, the Sheehan Disability Scale.

**TABLE 4:** The course of gambling disorder (abstinence, treatment status, participating in treatments, and self‐help groups) among outpatients at the follow‐up time points.

	With comorbidity (*n* = 35)	Without comorbidity (*n* = 25)	Time effect		Summary of significant differences	Time × group interaction	
			Wald *Χ* ^2^	*p*		*Wald Χ* ^2^	*p*
Gambling behaviors							
Abstinence (*n*, %)							
Baseline	26 (74.3)	24 (96.0)	9.490	**0.009**	*a* > c*, *b* > c*	2.384	0.304
3 months	22 (62.9)	18 (72.0)					
12 months	13 (37.1)	10 (40.0)					
Treatment status							
Still in treatment (*n*, %)							
3 months	20 (57.1)	15 (60.0)	5.683	**0.017**	*a* > b*	4.437	**0.035**
12 months	16 (45.7)	6 (24.0)					
Group meeting (*n*, %)							
3 months	15 (42.9)	10 (40.0)	7.701	**0.006**	*a* > b**	0.008	0.929
12 months	6 (17.1)	4 (16.0)					
Cognitive behavior therapy (*n*, %)							
3 months	4 (11.4)	5 (20.0)	0.432	0.511	*a* = *b*	0.685	0.408
12 months	5 (14.3)	3 (12.0)					
Counseling (*n*, %)							
3 months	8 (22.9)	5 (20.0)	1.630	0.202	*a* = *b*	0.589	0.443
12 months	6 (17.1)	2 (8.0)					
Social support							
Self‐help group (*n*, %)							
3 months	10 (28.6)	11 (44.0)	7.627	**0.006**	*a* > b**	0.474	0.491
12 months	3 (8.6)	2 (8.0)					

*Note:* *p* value for generalized estimating equations (*p < 0.05, **p < 0.01). Significant *p* values are shown in bold font (*p* < 0.05).

#### 3.2.1 Gambling Behavior

Patients with and without comorbidities exhibited significantly lower SOGS scores at 3 months and 12 months compared to baseline (each, *p* < 0.001, Table 3). In addition, a significantly lower proportion of both comorbid and non‐comorbid patients were abstinent from gambling at 12 months compared to baseline and 3 months (*p* = 0.009, Table 4). However, there were no significant changes in gambling activity (per the last 30 days) (Table 3) or for the Time × Group interaction effects for these outcomes (Tables 3 and 4).

#### 3.2.2 Social Impairment and Depressive Symptoms

Patients with and without comorbidity had significantly lower SDS work (*p* < 0.001), social or leisure activities (*p* = 0.007), home and family life (*p* < 0.001), and SDS total scores (*p* < 0.001), at 3 and 12 months as compared with baseline (Table 3). The PHQ‐9 scores were also higher at baseline than at 12 months (*p* = 0.014). In contrast, there were no significant Time × Group interaction effects for these outcomes.

#### 3.2.3 Treatment in Medical Institutions and Participation in Self‐Help Groups

A significant time effect was observed for still being in treatment at 12 months as compared with 3 months in patients with and without comorbidities (*p* = 0.017) (Table 4). A significant interaction between comorbidity status and time was also observed for being still in treatment. As compared with comorbid patients, a lower proportion of non‐comorbid patients was still in treatment at 12 months (*p* = 0.035) (Table 4).

As compared with the 3‐month time point, a significantly lower proportion of both comorbid and non‐comorbid patients participated in group meetings and self‐help groups at 12 months (each *p* = 0.006). However, there were no significant differences in treatment proportions for cognitive behavior therapy and counseling (Table 4).

## 4 Discussion

This study investigated the prognosis of multifaceted clinical problems (i.e., gambling problems, social function, depressive symptoms, treatment status, and social support) in GD outpatients in relation to comorbidity in clinical settings in Japan.

The present study found that multiple treatment outcomes such as SOGS scores and social dysfunction scores (i.e., in relation to work, family, and social functioning) decreased over time regardless of comorbidities. Some previous studies showed that patients with a GD who had comorbid psychiatric disorders (e.g., alcohol dependence and depressive symptoms) had a high relapse rate and a poor prognosis [3, 16, 24]. It is uncertain why the results from the current study contradict those from these previous studies, although it is possible that the clinical setting may be important in this context. Specifically, until now, the role of psychiatric comorbidity in the prospective course of GD during and after treatment has been primarily studied in inpatient settings. Previous research has shown that inpatients have more severe gambling problems and a higher proportion of comorbid psychiatric disorders such as anxiety and depression than those receiving outpatient services [21]. Thus, as inpatients have more severe problems than outpatients, it can be assumed that the characteristics of inpatients and outpatients are different. Indeed, a recent study of GD in an outpatient setting found that GD severity (the summed score of the endorsed DSM‐5 criteria) improved significantly in both outpatients with and without psychiatric comorbidity over the 3‐year observation period [32]. Given this, the results of this study suggest that in an outpatient setting there may not always be a significant difference between patients with or without comorbidity in terms of the clinical course of GD severity, GD problems, and in relation to social dysfunction.

One possible reason for why there was no significant difference in the study outcomes (e.g., gambling problems and social dysfunction) between patients with and without comorbidities was the higher rate of being still in treatment in patients with comorbidity. In this study, the still in treatment rate at 12 months declined sharply in the without comorbidity group from the 3‐month time point (60.0% → 24.0%), compared to in the with comorbidity group (57.1% → 45.7%), with a significant time × group interaction effect (*p* = 0.035). Given this, it can be speculated that receiving psychiatric treatment for comorbid conditions and/or GD might have an efficacious effect on each condition either directly and/or indirectly. An earlier mini review of psychiatric comorbidity in problem gambling [8] showed that lithium for comorbid bipolar disorder, escitalopram for comorbid anxiety, and the addition of CBT to standard drug treatment for comorbid schizophrenia may be effective, not only in decreasing the symptomatology of the comorbid psychiatric condition, but also in improving the gambling behavior [13, 17, 19, 29]. However, as yet, there is still little evidence that being treated for comorbid conditions might be actually beneficial for gambling‐related outcomes in individuals with GD, with several previous studies finding that treatment for psychiatric comorbidity had no effect on GD gambling problems [3, 16, 24]. Taken together, the results from the current and previous studies highlight the need for further research to clarify the effects of comorbidity treatments on gambling‐related activities and other outcomes in patients with GD.

This study has several limitations. First, the relatively small sample size was insufficient to enable a detailed examination of gambling‐related problems and clinical symptoms associated with GD across various comorbid psychiatric conditions. This limitation may have also increased the risk of type II errors. Second, the dropout rate during the follow‐up period reached 35.0%, potentially introducing attrition bias and compromising the robustness of the findings. Third, data on the total number of eligible patients during the study period were not collected, and participation was voluntary based on informed consent being obtained by medical staff. Consequently, no information was available regarding the characteristics of non‐participants, making it difficult to fully exclude the possibility of selection bias. Fourth, comprehensive screening for psychiatric comorbidities was not uniformly conducted for all participants, and information on comorbid conditions primarily relied on self‐reports and diagnoses provided by the doctor in charge. This reliance may have led to classification bias. Fifth, as various treatment outcomes were assessed using self‐report questionnaires, the possibility of socially desirable responding cannot be ruled out. Finally, this study was conducted between 2020 and 2023, during which time the COVID‐19 pandemic, particularly in its early phase, may have influenced healthcare‐seeking behaviors. In short, this study has a number of limitations that may have affected both the results obtained and the generalizability of our findings.

Despite these limitations, this study has several strengths: (1) it adopted a naturalistic prospective research approach for GD outpatients with psychiatric comorbidities in clinical settings in Japan, and (2) it investigated the course of multifaceted treatment outcomes between GD outpatients with and without psychiatric comorbidity in a clinical setting.

## 5 Conclusions

This study investigated the course of a multifaceted GD prognosis by comorbidity status in outpatients with GD. Contrary to our expectations, psychiatric comorbidity in outpatients with GD was not associated with poorer GD‐related outcomes when compared to these outcomes in GD outpatients without comorbidities. In addition, being still in treatment for comorbid psychiatric problems may affect the outcome of GD treatment and help protect against negative outcomes.

## Author Contributions

H.M. and R.Y. designed the study. H.M. and R.Y. recruited the patients and collected the data. R.Y. established a patient research database. Funding was obtained by H.M. and R.Y., H.M., A.S., and M.S. supervised the writing of the manuscript, the statistical analyses, and the initial draft manuscript was written by R.Y. All authors have revised and contributed to the writing of the final manuscript. All authors have read and approved the final manuscript for publication.

## Disclosure

Permission to Reproduce Material From Other Sources: Data available in article Supporting Information S1.

## Ethics Statement

This study was approved by the Ethics Committee at the Jikei University School of Medicine (31‐443 (10025)).

## Consent

Patients were given an information sheet about the study and asked to provide written informed consent before the data collection began.

## Conflicts of Interest

The authors declare no conflicts of interest.

## Supporting information


Data S1.

## Data Availability

The data are available in the article Supporting Information S1.
